# Editorial: Cosmeceuticals From Medicinal Plants

**DOI:** 10.3389/fphar.2020.01149

**Published:** 2020-07-29

**Authors:** Namrita Lall, Mohamad Fawzi Mahomoodally, Debora Esposito, Vanessa Steenkamp, Gokhan Zengin, Aimee Steyn, Carel B. Oosthuizen

**Affiliations:** ^1^ Department of Plant and Soil Sciences, Faculty of Natural and Agricultural Sciences, University of Pretoria, Pretoria, South Africa; ^2^ School of Natural Resources, University of Missouri, Columbia, MO, United States; ^3^ College of Pharmacy, JSS Academy of Higher Education and Research, Mysuru, India; ^4^ Department of Health Sciences, Faculty of Science, University of Mauritius, Réduit, Mauritius; ^5^ Department of Animal Science, North Carolina State University, Raleigh, NC, United States; ^6^ Department of Pharmacology, Faculty of Health Sciences, University of Pretoria, Pretoria, South Africa; ^7^ Department of Biology, Science Faculty, Selcuk University, Konya, Turkey

**Keywords:** cosmeceutials, medicinal plant, anti-inflammatory, acne (acne vulgaris), skin aging

The use of the word cosmetics comes from *kosmétikos*, an Ancient Greek term. This word can be translated as “skilled in adornment,” with the variant *kosmein* meaning “arrange” or “adorn” and *kosmos* meaning “order”: Further interpretations include “to make for beauty,” especially of the complexion, or beautifying and “done or made for the sake of appearance,” or “correcting defects especially of the face,” primarily it is “decorative” or “ornamental” (Oumeish, 2001). The concept of beauty is one of the aspects of the Greek word *kome*s, which means harmony, and was used to attain perfection. Gradually its meaning has changed until it became connected with the idea that was more closely related to the masking, concealing and camouflaging, as true beauty originates from the inner being and could not be created externally. Since primeval time, numerous civilisations have been subjected to the use of herbs as cosmetic applications. Even today, the demand and the utilization of phytocosmetics have increased in the personal care system (Mahomoodally and Ramjuttun, 2016). Research into the value and use of plant and mineral resources in cosmetics continued over the centuries evolving into what we consider to be cosmeceuticals. Interestingly, there is a great tendency of consumers to return to the use of herbs/herbal products for various applications to implement a more natural mode of life (Mahomoodally and Ramjuttun, 2016).

In a similar manner to “drugs,” defined as compounds utilized in the prevention and treatment of diseases, with the inherent potential to modulate physiological and pharmacological functions, so have “cosmetics” been designated as either cleaning or augmenting of skin appearance without therapeutic effects. The word “cosmeceuticals” has been coined to encompass that area which exists between the two aforementioned fields. Cosmeceuticals are hybrids that exist between drug and cosmetic products which are utilized to boost both skin health and beauty through their external and/or internal application. Natural cosmeceuticals include those elements that can have a medicinal effect on acne, pigmentary disorders, melasma (photoprotectants), aging, skin inflammation, wrinkle formation and scarring, as well as hair problems including thinning, and alopecia, to name but a few (Dorni et al., 2017). Resultantly, cosmeceutical research is an emerging field and aligns with health and economic challenges. This Research Topic included studies that provided scientific evidence for the advancement of phytochemicals and traditionally used plants as cosmeceuticals. It is anticipated that such studies will add to our understanding of the pharmacology and use of medicinal plants, fungi and other organisms used as cosmeceuticals through new and scientifically substantive knowledge.

The statement “there is a plant for every need on every continent,” now appears to be a truism (Cowan, 1999). Traditionally, the use of plants against skin diseases and specifically for cosmeceutical purposes has been a common practice among many cultures. A majority of the plant ingredients (oat, walnuts, chamomile, carrot, almonds, cucumber, lavender, mint, rose, and sweet violet petals) are utilized in modern phytocosmetics, including, shampoos, creams, lotions, and sun care products. Pieroni et al. (2004) documented that much of the external or topical applications of some of these plants have never been scientifically proven before, despite their ability to be used for either dermatological or cosmeceutical purposes.

This unique Research Topic comprises of 14 articles bringing together experimental and review papers on the pharmacology associated with these phyto-cosmeceuticals. The major themes within this topic included anti-acne, anti-inflammatory, pigmentary disorders, allergic reactions, aging, and repellent properties of medicinal plants.

Three book reviews have been included. The first book review; “Herbal Principles in Cosmetics: Properties and Mechanisms of Action” has as a topic a collection of medicinal plants that have been used traditionally as cosmeceuticals (De Canha et al.). The second book that was reviewed, “Cosmeceuticals and Active Cosmetics, Third Edition” provides a collection of medicinal plants that have been used in cosmeceuticals based on their active compounds. The book provides information on various ingredients and commercial products (De Canha et al.). The last book review; “Medicinal Plants for Holistic Healing,” includes a collection of medicinal plants that have been used as a remedy for treating various types of skin cancers, hyperpigmentation, oral disorders, and many others (McGaw et al.).

Sehlakgwe et al. focused on a plant, belonging to the Rosaceae family, which has previously been shown to have strong antibacterial properties against *Cutibacterium acnes*. The authors noted that significant differences were observed between the anti-acne activity of plant material collected in different seasons, with the best activity reported for plants collected during the winter period. Proton NMR-based untargeted metabolomic analysis was used to determine the differences in the chemical profiles of samples collected in different seasons. The compound 2-(4-ethoxyphenyl)-5,6,7,8-tetramethoxy-4H-1-benzopyran-4-one was identified in only the winter samples (Sehlakgwe et al.).

Pineau et al. investigated the inhibitory activity of *Callicarpa americana* leaves, a native South-Eastern United States shrub historically used by Native Americans to treat fever, stomach-ache, and pruritus against acne vulgaris skin condition. The latter is known to negatively affect adolescents and young adults by impacting self-esteem, self-confidence, and social life. This work highlighted the potential of the extracts as a cosmeceutical ingredient. The authors discussed the need for further research to assess its mechanism of action and *in vivo* efficacy (Pineau et al.).

Another study on ant-acne by De Canha et al. investigated the ability of methanolic extracts of *Helichrysum odoratissimum* to inhibit bacterial growth of *C. acnes* and pathogenic virulence factors thereof. The extract exhibited antimicrobial activity against *C. acnes* by showing high specificity against cell aggregation and preventing biofilm formation. Anti-inflammatory activity was observed by inhibiting cytokine levels of IL-8. This study validated the traditional use of *Impepho* as an ointment for pimples, not only by controlling bacterial proliferation but also due to inhibitory activity on various virulence factors (De Canha et al.).

The study by Ho et al. assessed the anti-inflammatory properties of spent coffee grounds (SCG) belonging to three Arabica cultivars. The extracts were found to exert inhibitory effects on the secretion of inflammatory mediators. Hawaiian Kona exhibited inhibitory effects on the expression of three examined cytokines, whereas Ethiopian Yirgacheffe reduced the secretion of TNF-α and IL-6, and Costa Rican Tarrazu decreased the secretion of IL-6. The anti-inflammatory activity was correlated with 26 identified metabolites quantified by LCMS. Twelve of these had high relative intensities in all of the extracts. The presence of multiple anti-inflammatory compounds in SCG provided a promising natural source for cosmeceutical and pharmaceutical industries (Ho et al.).

Fibrich et al. investigated how dermal aging is characterized by states of oxidative stress, chronic inflammation, and abnormal proteolytic degradation. This study focused on *Myrsine africana* and its active compounds, myrsinoside B and their ability to reduce the activity of hydrogen peroxide, superoxide and 5-lipoxygenase as supplementary mechanisms, ultimately to reduce the appearance of wrinkles. The samples showed the potential to be used as antiwrinkle agents as they indicated promising antioxidant and anti-inflammatory activity (Fibrich et al.).

Black walnut is known to be an excellent source of health-promoting and anti-inflammatory compounds. Ho et al. investigated ‘in-kernel’ extracts of Black walnut belonging to different cultivars and tested their effect on the expression of six human inflammatory cytokines/chemokines using a bead-based, flow cytometric multiplex assay. Certain black walnut cultivars showed promising potential for decreasing the severity of inflammatory skin diseases (Ho et al.).

Esposito et al. focused on Alaskan berries that have traditionally been used to treat skin wounds. The berries were found to contain a variety of bioactive polyphenols which exhibit anti-inflammatory, antioxidant, and antimicrobial properties, making them prime candidates for wound healing. Alaskan berries indicated promise and its usage in regenerative responses and restoring function in a variety of tissues and organs after injury or aging (Esposito et al.).

The paper by Hong et al. focused on atopic dermatitis (AD), of unknown origin. KHU-ATO-JIN-D (KAJD) which is a poly-herbal mixture of extracts from six plants, known to have anti-inflammatory and antiallergic effects, was found to inhibit the development of DNCB-induced AD in BALB/c mice and several immune cell types, suggesting that KAJD might be a useful therapeutic drug for the treatment of AD (Hong et al.).

Sim et al. reviewed the antiallergic potential of the family; Lamiaceae. This included its use as ethnomedicine for treating inflammatory skin diseases and allergic asthma and in-depth reference to the antiallergic mechanisms related to Lamiaceae species (Sim et al.).

Park et al. investigated the antimelanogenic effect of *Dendropanax morbiferus*. This plant was shown to inhibit tyrosinase activity and melanin formation by reducing melanogenesis-related protein levels. Two active ingredients of *D. morbiferus* were identified, namely, DMW-1 and DMW-2. It was concluded that *D. morbiferus* and DMW-1 may be useful as therapeutic agents for skin hyperpigmentation disorders (Park et al.).

The study by Narawi et al. focused on using nanoemulsions to decrease the volatility of the constituents of essential oils used as repellents. Different formulations were tested for their droplet size and insect repellent activity. A nutmeg oil-loaded nanoemulsion was successfully formulated and controlled release of the essential oil showed mosquito repellent activity, eliminating some of the disadvantages of crude essential oils (Narawi et al.).

These valuable scientific contributions are welcomed by the scientific community, however, the field of cosmeceutical research should be further expanded as one can tap into a wealth of discovery and development of important cosmeceuticals from natural resources to address consumer and patients demand.

## Author Contribution

All the guest editors and listed authors have made a direct contribution toward this work and approved it for publication.

## Conflict of Interest

The authors declare that the research was conducted in the absence of any commercial or financial relationships that could be construed as a potential conflict of interest.
